# Evaluation of Root Canal Obturation Quality in Deciduous Molars with Different Obturation Materials: An *In Vitro* Micro-Computed Tomography Study

**DOI:** 10.1155/2021/6567161

**Published:** 2021-07-02

**Authors:** Ayse I. Orhan, Esra C. Tatli

**Affiliations:** Department of Paediatric Dentistry, Faculty of Dentistry, Ankara Yıldırım Beyazıt University, Ankara, Turkey

## Abstract

**Objective:**

To evaluate the voids in root canal treatment of deciduous molar canals using three obturating materials and two obturation systems using micro-CT. *Study Design*. Thirty freshly extracted deciduous molars were used in this study. The specimens were instrumented using a ProTaper Universal rotary instrument and randomly assigned into six groups (*n* = 5). Mesiobuccal root canals were obturated using Ca(OH)_2_ and iodoform-Ca(OH)_2_ and ZOE cement. The materials were applied straight from the syringe up to the 2 mm coronal level of the apex. Subsequently, the Lentulo spiral and ultrasonic activation with endoactivator were used for obturation. All samples were scanned by micro-CT with 9.1 *μ*m isotropic voxel resolution. The voids in cross-sectional images and 3D volumes of voids were measured. Differences among materials were statically evaluated (*p* < 0.05).

**Results:**

All study groups showed voids. Ca(OH)_2_ and iodoform-Ca(OH)_2_ with ultrasonic activation produced fewer voids whereas the ZOE groups showed higher voids with statistical significance (*p* < 0.05).

**Conclusions:**

Ca(OH)_2_ and iodoform-Ca(OH)_2_ with ultrasonic activation decrease void formation. Further studies should be done with other obturation techniques and materials for deciduous tooth root canal management.

## 1. Introduction

The root canal treatment in pediatric endodontic treatment is to constitute the integrity and healthy relationship between the teeth and surrounding tissues while waiting for the deciduous teeth that are replaced by the permanent dentition [1]. The success in deciduous root canal treatment is being influenced by tight-sealed and 3D filling root canal to prevent residual bacteria from entering the periapical surrounding tissues with fewer voids as can be [2].

While performing the pulp therapy of deciduous teeth, some obstacles can affect the treatment such as root curve esp. in molar teeth, collateral canals, apical anatomy complexity, and probability of harm to underneath permanent teeth. These obstacles have initiated new obturation materials that could solve instrumentation problems and increase root canal treatment success [3]. Various obturation materials and techniques have been suggested for deciduous root canal treatment [4]. Zinc oxide, eugenol, calcium hydroxide, and iodoform-based pastes are being used in conjunction with a variety of obturation techniques, including endodontic pressure, premixed syringes, Lentulo spirals, and endodontic pluggers/reamers [5].

Ultrasound is commonly being used in endodontic procedures such as the coronal opening to periapical surgery [6]. The agitation of the irrigating solution induced by ultrasound improves its penetration of anatomically complex areas, such as the dentinal tubules, and so it improves cleaning [7]. The use of root canal filling materials promotes the penetration of dentin tubules and enhancing of sealing capacity [8] and antimicrobial activity [9]. However, the use of ultrasonic activation of the filling materials in deciduous canal management on the obturation quality is unclear.

Previous studies have evaluated the obturation quality of different obturation materials and techniques [10, 11]. However, computed tomography (CT) [10, 12] and the conventional radiographic method [11] are typically used to assess obturation quality. CBCT has a very low spatial resolution (100 to 1000 *μ*m), and radiographs are 2 dimensional. Moreover, the conventional radiographic method requires considerable time and is not standardized. Micro-CT is being applied for objective analysis of tooth structures which enables calculating and qualitative image analyses. It can also differentiate among obturation materials, voids, and tooth structures [13, 14].

No study has evaluated the effectiveness of ultrasound for deciduous root canal obturation using micro-CT. Hence, it was considered to assess the voids in deciduous molar canals using three obturating materials and two obturation systems by micro-CT. The null hypothesis is there would be no difference among techniques and materials in the obturation of the root canals.

## 2. Materials and Methods

The ethical approval was obtained from the Ankara University local ethical committee. Thirty deciduous second molar teeth that were extracted after clinical and radiographic diagnoses with carious lesion were used. The teeth had no root resorption. The teeth were randomly distributed into six groups (*n* = 5). Access opening was performed using a high-speed no. 330 diamond bur with a copious water supply.

### 2.1. Root Canal Preparation

The mesiobuccal canals were prepared until F3/0.06 using ProTaper Universal (Dentsply Sirona, Canada). 1% NaOCl was applied to the canals between instrumentation following 17% EDTA for the final irrigation. Then, they were dried using sterile paper points (Pearl Dent Co., Ltd., Ho Chi Minh City, Vietnam).

### 2.2. Root Canal Obturation

Obturation was performed using the following three materials:

1. Zinc oxide eugenol cement (ZOE; Master-Dent, Dentonics, Inc., Monroe, LA) mixed (powder : liquid ratio of 1 : 2) on a glass slab; the cement was administered directly from the syringe up to 2 mm coronal level of the apex
2. Calcium hydroxide (Ca(OH)_2_) (Metapaste, Meta Biomed, Chungbuk, South Korea)
3. Iodoform-Ca(OH)_2_ (Metapex, Meta Biomed, Chungbuk, South Korea) was applied to the root canals straight from the prepacked syringe up to 2 mm coronal to the apex

### 2.3. Obturation Techniques

The specimens were distributed into 6 groups as follows: group I: ZOE+Lentulo spiral, group II: ZOE+ultrasonic activation, group III: Ca(OH)_2_+Lentulo spiral, group IV: Ca(OH)_2_+ultrasonic activation, group V: iodoform-Ca(OH)_2_+Lentulo spiral, and group VI: iodoform-Ca(OH)_2_+ultrasonic activation.

Lentulo spirals and ultrasonic activation were used as obturation techniques. In the Lentulo groups, a size 25 Lentulo spiral (Golden Star Medical Co., Ltd., Guangdong, China) was inserted in a low-speed handpiece (20.000 rpm). The Lentulo spiral was then applied to the canal in a clockwise direction rotation and taken out from the canal smoothly while still rotating. The procedure was repeated approximately for 10 s until backfill was seen in the canal orifice.

In the ultrasonic activation groups, an endoactivator (25 medium tips) (Dentsply Sirona, Canada) in combination with a Waterpik Fla-220 power flosser (Teledyne Waterpik, Fort Collins, Colorado) was used during 20 s. The procedure was applied at least twice until backfill was seen in the orifice. For each sample, when canals were assumed to be filled, a wet cotton pellet was used to lightly tamp the material into the canals.

### 2.4. Micro-CT

The teeth were scanned using a high-resolution, desktop micro-CT system (Bruker Skyscan 1275, Kontich, Belgium). The scanning parameters were 100 kVp, 100 mA beam current, 0.5 mm Al/Cu filter, 9.1 *μ*m pixel size, with 0.5 rotation steps in 360° rotation. To overcome artifacts, various filters were applied such as air calibration of the detector was carried out for reducing the ring artifacts. The approximate scanning duration was around 1 h.

Other settings were used according to the manufacturer's instructions such as beam-hardening correction, based on scout scanning.

### 2.5. Micro-CT Image Analyses

The NRecon software (ver. 1.6.7.2, Skyscan, Kontich, Belgium) and CTAn (ver. 1.17.7.2, Skyscan) were used for visualization and quantitative measurement of the samples using the modified algorithm of Feldkamp et al. to generate axial, two-dimensional, 1000 × 1000 pixel images [15]. In terms of reconstruction parameters, ring artifact correction and smoothing were fixed at zero, and beam artifact correction was set at 40%. Contrast limits were applied following Skyscan's instructions. Using the NRecon software (Skyscan, Kontich, Belgium), the images were reconstructed to show two-dimensional slices of the roots. In total, 890 cross-sectional images were reconstructed from the whole volume. The CTAn (Skyscan, Aartselaar, Belgium) software was used for three-dimensional volumetric visualization, analysis, and measurement of the volume of the root canal.

The presence of voids was assessed in two-dimensional slices of each section following Orhan et al.'s study [16]. New cross-sectional images were prepared perpendicular to the long axis of the root, starting at the most apical part of the root. The sections had an interval of 0.5 mm, resulting in approximately 250 axial images. All images of the sections were transformed into. tiff files. Two observers independently from each other examined all sections using a binary registration scale for voids. The observers were free to change the optimization filters, and also, those observers were blinded to the root-obturation techniques. For disagreement cases between the observers, the sections were reevaluated, until a consensus was reached.

**Figure 1:**
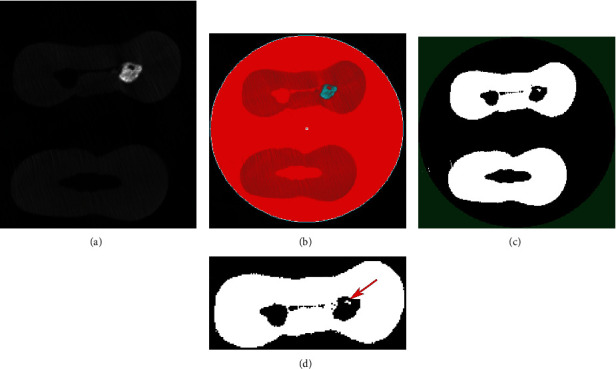
Axial micro-CT images showing (a) obturated mesiobuccal canal, (b) ROI for measurements, (c) binary registration scale for canals, and (d) white pixels in the canal showing void (arrow).

**Figure 2:**
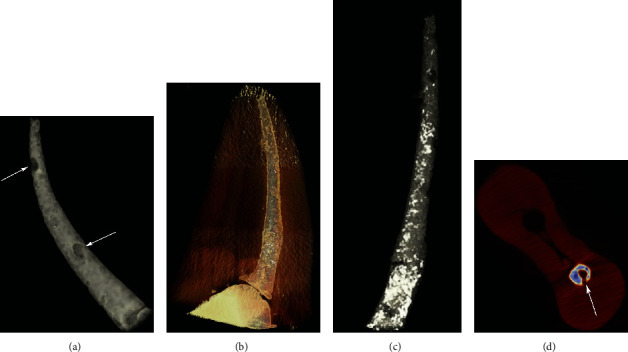
(a) 3D representation of the root canal obturation with voids. (b) 3D representation showing the root and canal obturation. (c) Image showing voids in white color. (d) Axial image showing voids to canal wall (arrow).

A Gaussian low-pass filter for noise reduction was used to calculate voids in 3D volumes. The images were sorted out with a global segmentation threshold to subtract dentin from sealer and voids using the CTAn software. A binarization process was applied, to generate an image of black/white pixels. Subsequently, each slice region of interest was traced to contain a single object entirely to allow the calculation of void volumes (Figure 1). 3D visualization and evaluation of canal obturations were done using the CTVox software (version 3.3.0, Bruker micro-CT) (Figure 2).

The mean obturation percentage of the root canal in 2D slices and volume of voids distributed along with materials in 3D volume were calculated by micro-CT analyses.

### 2.6. Statistical Analyses

The data were analyzed using SPSS (ver. 22; Chicago, IL, USA). Interexaminer reliability of the presence and absence of obturation and void existence was evaluated using Cohen's kappa statistical analysis. Pairwise comparisons were performed using the Mann–Whitney *U* test and Kruskal–Wallis *H* test with the level of significance set at *p* < 0.05.

## 3. Results

The interexaminer reliability showed a high agreement of 88.2% (95% CI 0.7542–0.851) between the two observers (KO, BC) which showed no significant difference between observers.

The results are shown in Tables 1 and 2. The mean obturation percentage of the canal in 2D slices that were obturated with Ca(OH)_2_+ultrasonic activation (group IV) and iodoform-Ca(OH)_2_+ultrasonic activation (group VI) had a significantly better obturation percentage than the other groups (*p* < 0.05). On the other hand, no significant differences were found between these groups (*p* > 0.05).

The mean volume of the voids in 3D volumes showed no significant difference among groups I, II, III, and V (*p* > 0.05). There were fewer voids for groups IV and VI than the other groups (*p* < 0.05). On the other hand, again, no significant differences were noted between these groups (*p* > 0.05).

## 4. Discussion

Caries lesions in deciduous molars progress faster than those in other deciduous teeth because of their occlusal morphology [17], and the pulp may be involved easily. Deciduous teeth diagnosed with irreversible pulpitis or necrosis can be treated effectively with pulpectomy. The achievement of a good root canal treatment in deciduous teeth depends on the quality of biomechanical preparation, type of obturating material used with as few voids as possible, and achievement of a good hermetic seal [11, 18].

To date, no material has been shown to own the essential properties of an ideal root canal obturation material for deciduous teeth. ZOE paste, calcium hydroxide, and iodoform paste are the most commonly used materials [19].

Although ZOE has some drawbacks such as slow resorption, periapical tissue disruption, necrosis of bone, and cementum among others [20], it is one of the most commonly used pastes. The use of ZOE was recommended in 2008 by the American Academy of Pediatric Dentistry (AAPD) for the treatment of deciduous teeth. Ca(OH)_2_, a silicone oil-based paste, is also widely used for its antibacterial, resorbable, and biocompatible features [21]. Furthermore, iodoform plus Ca(OH)_2_ is being used due to its antibacterial effect, healing properties, and ability to be resorbed when used in excess [22, 23]. Therefore, in this study, these three obturation materials with two obturation techniques (Lentulo spiral and ultrasonic) were compared.

Premixed pastes exhibited better performance compared to ZOE (*p* < 0.05), likely because they are premixed, which eliminates problems caused by mixing and manipulation. Moreover, premixed pastes can be administered into the narrow and tortuous root canals of deciduous molars and reach the apex or beyond because of their followability. ZOE is not a premixed material, and so its consistency depends on clinician experience and manipulation. In this study, ZOE with a powder/liquid ratio of 1 : 2 was used to obtain a creamy consistent material.

Lentulo spiral enables endodontic delivery of the root canal sealer [5] but requires a high level of operator skill. Even experienced operators may need to reinsert the material to ensure good obturation quality that may increase the treatment time [19]. In some studies, Lentulo spiral is reportedly superior to the syringe or needle technique [10, 24, 25]. However, others reported similar results for delivering paste into the root canal [26, 27]. On the other hand, ultrasound enables the delivery of obturation material and the placement of mineral trioxide aggregate (MTA) into the curved canals. Ultrasonic activation has been suggested to improve the physical properties of MTA, including compressive strength and microhardness [28]. Keles et al. used two MTA placement techniques, for orthograde obturation of the mesial root canals of mandibular first molars [29]. They stated no significant differences between manual compaction and ultrasonic activation of manually compacted MTA techniques for placement of MTA.

In this study, obturation quality did not significantly differ between ultrasonic and Lentulo spiral obturation of root canals (*p* < 0.05). Although ultrasound is not frequently used to deliver the obturation materials to root canals, this study proposed that the utility of the ultrasonic obturation technique can be beneficial for the obturation of deciduous molars with less void formation. However, further studies should be done for this technique with other obturation materials in comparison to the operative experience.

## 5. Conclusion

As a consequence of the limitations of this study, the incidence of voids within root canal obturations can be influenced by clinician experience, manipulation, obturation technique, mixed or premixed sealer, and the anatomical configuration of the canal. The ultrasonic technique can enhance the quality of root canal obturation and decrease the void volume.

## Figures and Tables

**Table 1:** Table showing the mean percentage (standard deviation) of the obturation percentage of the root canal in 2D slices. Bold numbers show statistical significance.

Groups	*n*	Mean (range)	SD	Statistical test	
				Pairwise comparison	*p* value
Group I ZOE+Lentulo spiral	5	83% (26.3-100)	±0.6	1-4	*p* > 0.05
				1-6	

Group II ZOE+ultrasonic activation	5	86% (29.2-100)	±0.5	2-4	*p* > 0.05
				2-6	

Group III Ca(OH)_2_+Lentulo spiral	5	88% (24.7-100)	±0.3	3-4	*p* > 0.05
				3-6	

Group IV Ca(OH)_2_+ultrasonic activation	5	94% (25.5-100)	±0.4	1-4	^∗^ **p** < 0.05
				2-4	
				3-4	
				4-5	

Group V Iodoform-Ca(OH)_2_+Lentulo spiral	5	89% (24.3-100)	±0.5	5-4	*p* > 0.05

Group VI Iodoform-Ca(OH)_2_+ultrasonic activation	5	94% (28.7-100)	±0.3	1-6	^∗^ **p** < 0.05
				2-6	
				3-6	
				5-6	

**Table 2:** Mean volume (standard deviation) of voids of the root canal obturation materials in 3D volumes. Bold numbers show statistical significance.

Groups	*n*	Mean (range) (mm^3^)	SD	Statistical test	
				Pairwise comparison	*p* value
Group I ZOE+Lentulo spiral	5	3.116	±0.125	1-4	*p* > 0.05
				1-6	

Group II ZOE+ultrasonic activation	5	2.725	±0.145	2-4	*p* > 0.05
				2-6	

Group III Ca(OH)_2_+Lentulo spiral	5	2.618	±0.214	3-4	*p* > 0.05
				3-6	

Group IV Ca(OH)_2_+ultrasonic activation	5	2.217	±0.195	1-4	^∗^ **p** < 0.05
				2-4	
				3-4	
				4-5	

Group V Iodoform-Ca(OH)_2_+Lentulo spiral	5	2.415	±0.218	5-4	*p* > 0.05

Group VI Iodoform-Ca(OH)_2_+ultrasonic activation	5	2.223	±0.174	1-6	^∗^ **p** < 0.05
				2-6	
				3-6	
				5-6	

## Data Availability

All data of the present article are available on request by contacting the corresponding author.
